# *Morinda Officinalis* Polysaccharides Stimulate Hypothalamic GnRH Secretion in Varicocele Progression

**DOI:** 10.1155/2017/9057959

**Published:** 2017-09-20

**Authors:** Zhu Zhu, Feng Huang, Feng Wang, Yonghong Zhang, Xiaozhen Zhao, Wei Wang

**Affiliations:** ^1^Department of Human Anatomy and Histoembryology, School of Basic Medical Sciences, Fujian Medical University, Fuzhou 350122, China; ^2^Key Laboratory of Brain Aging and Neurodegenerative Diseases of Fujian Provincial Universities and Colleges, Fuzhou 350122, China; ^3^School of Pharmacy, Fujian Medical University, Fuzhou 350122, China; ^4^Research Center for Neurobiology, School of Basic Medical Sciences, Fujian Medical University, Fuzhou 350122, China

## Abstract

Varicoceles (VCs) are the predominant cause of male infertility and are a risk factor for chronic venous disease.* Morinda officinalis* (*M. officinalis*) is a traditional Chinese medicine used to tonify the kidney and strengthen yang. In this study, we evaluated the effects of water-soluble polysaccharides extracted from* M. officinalis* (MOPs) on gonadotropin-release hormone (GnRH) secretion in a classic experimental left VC (ELV) rat model. Intragastric administration of MOPs at a dose ranging from 50 mg kg^−1^ to 100 mg kg^−1^ facilitated improvements in sperm parameters and seminiferous epithelial structures, modulated serum hormone profiles, and stimulated GnRH synthesis and release in the hypothalamus. MOPs also promoted spinogenesis and functional spine maturation in the arcuate nuclei (Arc), wherein they acted mainly on Kiss1 and GnRH neurons. Moreover, MOP-mediated Kisspeptin-GPR54 pathway upregulation and MAPK phosphorylation activation may have been responsible for increases in GnRH synthesis and release. Collectively, the findings of this study indicate that MOPs were effective in stimulating GnRH secretion, possibly by upregulating the Kiss1/GPR54 pathway and enhancing synaptic plasticity, and that MOPs can serve as a therapy for early VCs.

## 1. Introduction

Varicoceles (VCs), which are defined as dilations and tortuosities of the pampiniform venous plexus in the spermatic cord, are the predominant cause of male infertility [1]. VCs have been associated with testicular discomfort and atrophy and may be a risk factor for hypogonadism [2, 3]. VCs are characterized by the following histopathological changes: degenerative changes in the germinal epithelium and increases in cell apoptosis [4]. Left-sided VCs comprise 85–90% of cases as a result of venous congestion and rigid posture [3, 5]. Experimental left varicocele (ELV) models using dogs and rats are the most commonly used classic models that mimic VC progression in men [6]. ELVs usually lead to degeneration of the seminiferous epithelium at least four weeks after their establishment via surgery [7, 8].

VCs have not been detected in any children <10 years old [9]. However, the prevalence of VCs in boys aged 10–19 years was 16.2%, a percentage similar to those reported in new military recruits [10–12] and fertile men presenting for vasectomy [13]. The incidence of VC has been shown to increase by approximately 10% in each successive decade of life and to reach as high as 75% after the eighth decade of life [14]. The severities of spermatogenic arrest and hypospermatogenesis have been reported to be correlated with VC duration [15]. Additionally, the results of a prospective cohort study suggested that VCs are a risk factor for inguinal hernias in younger patients and chronic venous disease in older patients [16]. Thus, treating VCs during their early phases and preventing them in high-risk groups of patients are essential for preventing VC-related injuries.

*Morinda officinalis* (*M. Officinalis*, Bajitian in Chinese, family Rubiaceae), the dried root of a vine grown in southeast China, is a type of traditional Chinese medicine used to treat many diseases, including impotence, menstrual disorders, depression, osteoporosis, and inflammation [17–24], and is listed in the Chinese Pharmacopoeia [25]. The water layer of the aqueous extract of* M. officinalis* has been reported to be effective in promoting spermatogenesis in injured testes [26, 27].* M. officinalis* polysaccharides (MOPs), which are important constituents of the roots of* M. Officinalis*, are water-soluble and exist mainly in water extracts of* M. officinalis*. The results of our previous study suggested that MOPs promoted spermatogenesis and increased serum gonadotropin-releasing hormone (GnRH) levels in experimental VCs [28], indicating that MOPs may be effective in regulating hypothalamic GnRH pulses. Thus, the objective of this study was to determine the effects of MOPs on GnRH synthesis in VC progression and to elucidate the mechanisms underlying these effects to determine whether MOPs can be used to treat or prevent VCs.

GnRH neurons, which are scattered throughout the anterior region of hypothalamus and project to the median eminence (ME), release GnRH, the major neurohormone involved in vertebrate reproduction, as the final output signal in the process of vertebrate reproduction [29]. In males, GnRH is responsible for stimulating the secretion of luteinizing hormone (LH) and follicle-stimulating hormone (FSH), followed by the secretion of testosterone (T) [30, 31].

This study was designed to investigate the effects of MOPs on GnRH secretion and regulation in ELV rats. The arcuate nuclei (Arc) and ME expression levels of GnRH, Kiss1, and GPR54, as well as the phosphorylation of mitogen-activated protein kinases (MAPKs) and crucial GnRH transcription factors, were evaluated, and the spines of neurons in the Arc were observed to determine whether MOPs promote spinogenesis in VCs.

## 2. Materials and Methods

### 2.1. Extraction and Purification of MOP

Dried* M. officinalis* roots were purchased from a local herb market, and MOPs were extracted via water extraction and alcohol precipitation [32], as previously described [28].

### 2.2. Animals and Groups

Eighty-four mature male Sprague-Dawley (SD) rats weighing 200 ± 20 g were obtained from the Laboratory Animal Center of Fujian Medical University, China (No. SCXK(Min)2012-0001). The animals were maintained one per cage and at a constant temperature (22 ± 2°C) and humidity (50 ± 10%) under a 12 h light/12 h dark cycle during the study. The animal experiments were performed in accordance with the guidelines of the local ethics committee (No. 2015-29), and the animals were handled in accordance with the Guide for the Care and Use of Laboratory Animals.

The rats were randomly divided into the following seven groups (*n* = 12 per group): a sham-operated group (SO group), an ELV model group (ELV group), and five ELV model groups treated with double-distilled water (solvent control group, SC group), 25 mg kg^−1^ MOP (M25 group), 50 mg kg^−1^ MOP (M50 group), 100 mg kg^−1^ MOP (M100 group), and 200 mg kg^−1^ MOP (M200 group), respectively. The ELV rat model was established as described by Saypol et al. [6]. The rats in the SO group underwent a similar operation; however, no vessels were ligated. Three days after surgery, the rats were intragastrically administered either pure water or one of the four indicated doses of MOP for 4 weeks. A particular animal was included in the following studies only if the diameter of the ipsilateral spermatic vein, that is, the spermatic vein on the side of the surgery, was more than 3 times the diameter of the contralateral (healthy) spermatic vein, and renal atrophy did not occur.

### 2.3. Sperm Analysis

The left cauda epididymis was minced in 2 ml of cold phosphate-buffered physiological saline (PBS, pH = 7.2), and an aliquot (0.05 ml) of the resulting sperm suspension, which was filtered using an 80 *μ*m nylon mesh, was diluted and mixed well with PBS (1 : 40). A sample of the diluted sperm suspension was then loaded into a hemocytometer, and the sperm count across 8 squares (1 mm^2^ each) was determined and multiplied by 5 × 10^6^ to represent the number of sperm per epididymis. Additionally, smears prepared with the diluted sperm supernatant were fixed with 10% formaldehyde and then stained with 5% eosin before being observed microscopically. According to the criteria developed by Wyrobek and Bruce [33], 300 sperms per animal were assessed for morphological abnormalities. All the tests were conducted independently by two observers.

### 2.4. Serum Hormones

Blood samples were collected and allowed to clot overnight at 4°C before being centrifuged for 20 minutes at 1000*g*. The supernatant was subsequently collected, and enzyme immunoassays (EIAs) for GnRH (S-1217, LHRH-EIA Kit, Peninsula Laboratories International Inc., San Carlos, CA, USA), LH (LH ELISA Kit, ENZ-KIT107, Enzo Life Sciences Inc., Berne, Switzerland), FSH (Rat FSH ELISA Kit, abx051243, Abbexa, Cambridge, UK), and testosterone (T) (Testosterone ELISA Kit, ADI-900-065, Enzo Life Sciences Inc.) were conducted.

### 2.5. Histopathological Staining

The left testicle of each mouse was embedded in OCT compound, and 20 *μ*m tissue slices were produced using a cryostat. These slices were stained with hematoxylin and eosin (H&E) [34]. Testicular injury and spermatogenesis were graded with Johnsen's score (JS). Specifically, 300 seminiferous tubules from each animal were observed, and each was given a score of 1 to 10 [35].

Nissl staining (methylene blue) was performed on slides containing frozen tissues from the hypothalamus [36]. Five slides from each animal were observed under 200x magnification, and the total numbers of neurons in the Arc were counted. Golgi-Cox staining of the Arc was conducted as previously described [37]. The neurons in the Arc were imaged with an inverted light microscope (Ti-s, Nikon Corporation, Tokyo, Japan), and neurons with unobstructed dendrites along their entire length were chosen for analysis. The spines were morphologically characterized under 400x magnification and were classified as thin (filopodial and thin spines) and large spines (mushroom, stubby, and fenestrated spines) [38]. The percentages of spines with each shape were calculated. Moreover, the spine density was calculated as the number of spines per 50 *μ*m of dendrite length. All the slides were independently observed, imaged, and assessed by two investigators.

### 2.6. Immunofluorescence

Serial hypothalamic sections (10 sections per sample) were blocked with 10% donkey serum and then incubated with antibodies against GnRH (dilution 1 : 100, sc-32292, Santa Cruz Biotechnology Inc., Dallas, TX, USA), Kiss-1 (dilution 1 : 100, sc-18134, Santa Cruz Biotechnology Inc.), and GPR54 (dilution 1 : 100, sc-134499, Santa Cruz Biotechnology Inc.) for 24 h at 4°C. The sections were then treated with the following secondary antibodies: Alexa 488-conjugated donkey anti-mouse IgG (A-21202, Thermo Fisher Scientific Inc., Waltham, MA, USA), Alexa 633-conjugated donkey anti-goat IgG (A-21082, Thermo Fisher Scientific Inc.), and Alexa 594-conjugated donkey anti-rabbit IgG (A-21207, Thermo Fisher Scientific Inc.). After being stained with DAPI (D1306, Thermo Fisher Scientific Inc.), the sections were imaged and analyzed with a laser scanning confocal microscope (SP8, Leica Microsystems Inc., Buffalo Grove, IL, USA).

### 2.7. In Situ Hybridization

In situ hybridization was used to detect GnRH mRNA expression in the hypothalamus (In Situ Hybridization Kit for GnRH, MK2183, Boster, Wuhan, China). Five consecutive sections from each animal were chosen for analysis, and the number of immunoreactive neurons in the Arc was determined by two observers.

### 2.8. Western Blot Analysis

Using the rat brain atlas, we located the arcuate nucleus (Arc), including the ME, and micropunched it with spinal needles (0.8 mm internal diameter). The total protein was collected, and the protein concentrations were determined with a BCA Protein Assay Kit (Beyotime Institute of Biotechnology, Shanghai, China). Thirty micrograms of total protein was loaded onto a 16% Tricine-SDS-PAGE gel and then transblotted onto a polyvinylidene fluoride membrane (ISEQ00010, Millipore Inc., Boston, MA, USA), which was subsequently incubated with antibodies against GnRH, p38 (ab 182453, Abcam Inc., Cambridge, MA, USA), phospho-p38 (phospho-T180 + phospho-Y182; ab 45381, Abcam Inc.), p44/42 (#4695, Cell Signaling Technology, Inc., Beverly, MA, USA), phospho-p44/42 (#4370, Cell Signaling Technology, Inc.), synaptophysin (ab32127, Abcam Inc.), and GAPDH (ab 8245, Abcam Inc.). After the membrane had been incubated with horseradish peroxidase-conjugated secondary antibodies (31466, 32230, Thermo Fisher Scientific Inc.), the resulting bands were visualized with ImageQuant LAS 4000 mini (General Electric Company, Fairfield, CT, USA) with ECL Plus Western blotting substrate (Beyotime Institute of Biotechnology), and the integrated intensities of the bands were measured by Image J. The results are expressed as the relative integrated intensity minus the background intensity, which was represented by the expression of GAPDH.

### 2.9. Quantitative Real-Time RT-PCR

Total RNA from the Arc was extracted with Trizol and converted to cDNA using a PrimeScript RT Reagent Kit (RR047A, Takara Bio Inc., Dalian, Shandong, China), according to the manufacturer's instructions. Real-time PCR was performed with primers and cDNA templates mixed with SYBR Premix Ex Taq II (DRR081, Takara Bio Inc.). The gene-specific primers (shown in Table 1) were synthesized by Sangon Biotech (Shanghai, China), and the transcript levels of each gene were normalized to those of GAPDH in the same sample. The data were analyzed and quantified using the ΔΔCt method.

### 2.10. Statistical Analysis

The intensities of the fluorescence within the Arc and ME samples were measured using Image Pro Plus software version 6.0 (Media Cybernetics Inc., Rockville, MD, USA), and the results were analyzed using SPSS software version 22.0 (SPSS Inc., Chicago, IL, USA). Normally distributed continuous data (tested with the one-sample Kolmogorov-Smirnov test) were expressed as the mean ± standard deviation (SD). Differences among the groups were analyzed by one-way analysis of variance (ANOVA), after which the differences between two groups were analyzed by the least significant difference test. *P* < 0.05 was considered statistically significant.

## 3. Results

### 3.1. Parameters of the Sperm in the Ipsilateral Cauda Epididymis

**Figure 1:**
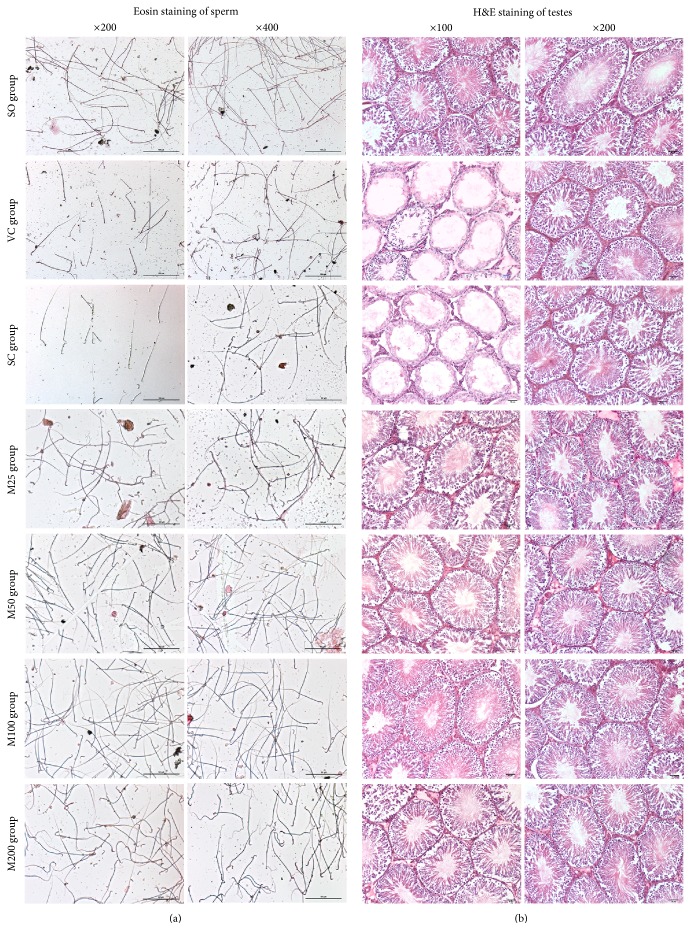
Sperm smears stained with 1% eosin (a) and HE-stained images of the testes (b) in the experimental groups.

The sperm count in the left epididymis of the ELV group was significantly decreased compared with that of the SO group (*P* < 0.05), and a higher percentage of sperm morphologic abnormalities were observed in the ELV group than in the SO group (*P* < 0.05). The sperm counts of both the M50 group and the M100 group were significantly increased compared with those of the ELV group (*P* < 0.05), and the percentages of sperm with morphologic abnormalities were significantly decreased in the former groups compared with the latter group (*P* < 0.05). The sperm count of the M200 group was also significantly increased compared with that of the ELV group (*P* < 0.05), and the percentage of sperm with morphologic abnormalities was significantly elevated in this group compared with the SO group (*P* < 0.05) (Figure 1(a), Table 2).

### 3.2. Ipsilateral Testis Morphology

HE staining (Figure 1(b)) showed that normal seminiferous tubules with normal interstitia were present in the testicular sections from the SO group. In contrast, the interstitial spaces of the testicular sections from the ELV group were wide and loose. Moreover, the tubules of the ipsilateral testes from the ELV group displayed a severely damaged seminiferous epithelium and a significantly decreased number of spermatogenic cells, as well as interstitial necrosis. After MOP treatment, the histology of the seminiferous epithelium improved, as treated tissues displayed more intact seminiferous tubules, which contained larger numbers of organized spermatogenic cells and larger numbers of sperm than ELV tissues. The testes in both the M50 group and the M100 group displayed relatively normal seminiferous tubules and interstitia.

JS scores, which were assigned according to the Johnson criteria (Table 2), were significantly decreased in the ELV group compared with the SO group (*P* < 0.05). MOP treatments significantly increased JSs in the corresponding groups compared with the ELV group (*P* < 0.05), and the JSs of the M100 group were similar to those of the SO group.

### 3.3. Changes in GnRH Secretion and Kiss1-GPR54 Pathway Activity

**Figure 2:**
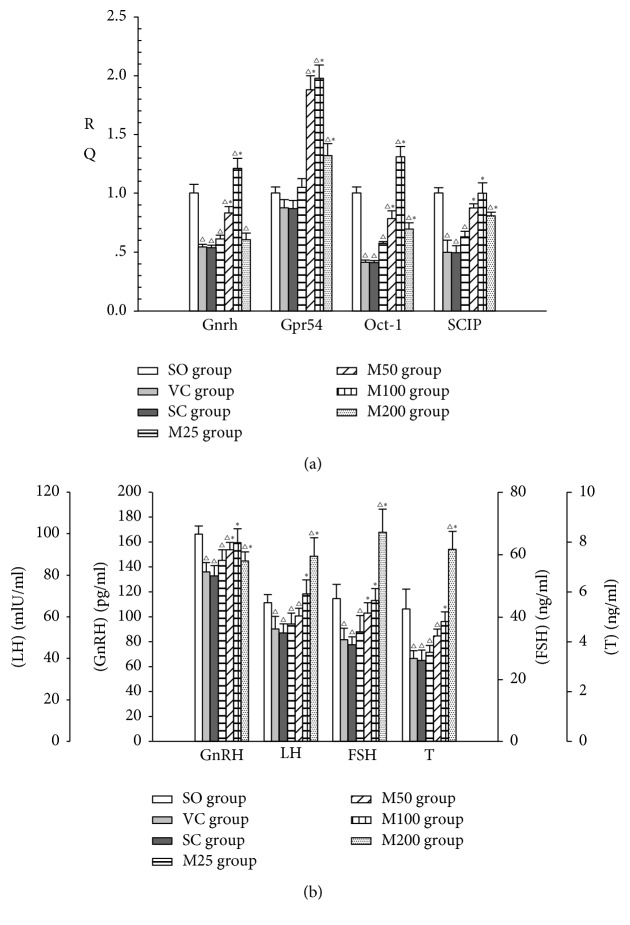
GnRH, GPR54, and GnRH-related transcription factor levels in the Arc (a) and the serum hormone profiles (b) of the experimental groups. △: *P* < 0.05: compared with SO group; *∗*: *P* < 0.05: compared with VC group.

As shown in Figure 2(b), after surgery, serum GnRH, gonadotropin and T levels were significantly decreased in the ELV group compared with the SO group (*P* < 0.05). However, treatment with 50 mg kg^−1^ MOPs significantly increased serum GnRH and FSH levels (*P* < 0.05), and treatment with 100 mg kg^−1^ MOPs significantly increased serum GnRH, gonadotropin, and T levels in the corresponding groups compared with the SO group (*P* < 0.05). The rats in the M200 group displayed significantly increased serum gonadotropin and T levels (*P* < 0.05) but decreased serum GnRH levels compared with the rats in the SO group.

**Figure 3:**
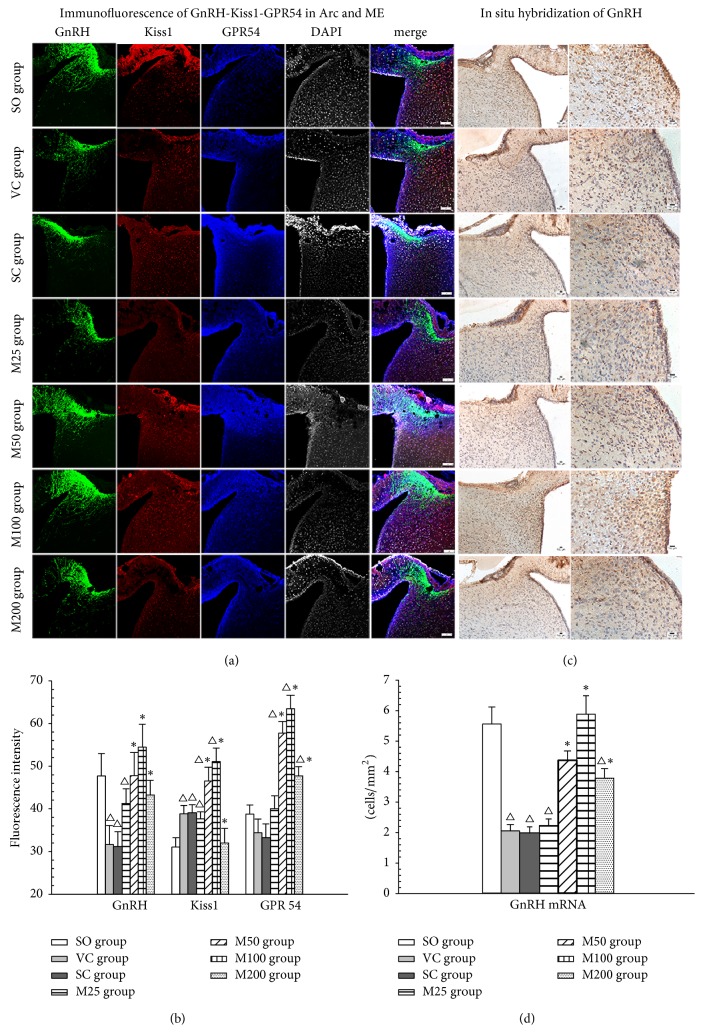
Effects of MOPs on GnRH section and the Kiss1/GPR54 pathway in the Arc. The effects of MOPs on GnRH, Kiss1, and GPR54 expression in the Arc of ELV rats (a). The fluorescence intensities of GnRH and Kiss1/GPR54 in the Arc of the experimental groups (b). MOPs increased the number of neurons that stained positive for GnRH mRNA expression in the Arc of the ELV group (c). The number of neurons exhibiting GnRH mRNA immunoreactivity in the Arc of the experimental groups (d). △: *P* < 0.05: compared with SO group; *∗*: *P* < 0.05: compared with VC group.

As shown in Figure 3(a), GnRH immunoreactivity was located mainly in the lateral portion of the Arc and the internal layer of the ME, while Kiss-1 and GPR54 immunoreactivity was located mainly in the medial portion of the Arc. The fluorescence intensities of Kiss1, GPR54, and GnRH in the Arc and ME of the hypothalamus were significantly decreased in the ELV and SC groups compared with the SO group (*P* < 0.05). The fluorescence intensities of GnRH, Kiss1, and GPR54 were significantly increased in the M50 and M100 groups (*P* < 0.05) but decreased in the M25 and M200 groups compared with the SO group. Moreover, Kiss-1 and GPR54 expression levels in the M200 group were significantly lower than those in the M100 group (*P* < 0.05).

The in situ hybridization results (Figures 3(c) and 3(d)) showed that positive staining for GnRH mRNA was localized in the endochylema and nuclei of neurons in the Arc. The numbers of neurons expressing GnRH mRNA were significantly decreased in the ELV and SC groups compared with the SO group (*P* < 0.05). However, the number of GnRH-positive neurons was significantly increased in the M50 and M100 groups compared with the ELV group (*P* < 0.05).

**Figure 4:**
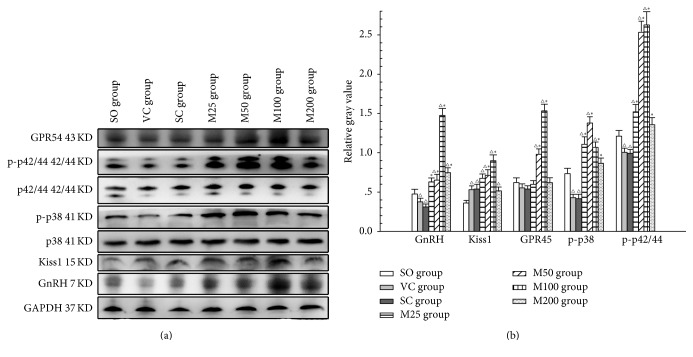
GnRH and Kiss1/GPR54 pathway activity in the Arc of the experimental groups. (a) Western blot results showing that MOPs upregulated Kiss1 and GPR54 expression, as well as the phospho-MAPK-to-total MAPK ratio, in the hypothalamus of ELV rats. (b) The relative gray value of the proteins in the Arc of the experimental groups. △: *P* < 0.05: compared with SO group; *∗*: *P* < 0.05: compared with VC group.

The western blot results (Figure 4) showed that the ratios of phospho-p42/44 and phospho-p38 to total p42/44 and p38, respectively, were significantly decreased in the ELV and SC groups compared with the SO group (*P* < 0.05). However, the levels of these proteins were significantly increased in the M50 and M100 groups compared with the ELV group (*P* < 0.05). The increases in hypothalamic GnRH and MAPK expression levels in the M200 group were less pronounced than those in the M100 group (*P* < 0.05).

Oct-1, Oct-6, and GnRH mRNA expression levels were significantly decreased in the ELV group compared with the SO group (*P* < 0.05) and were significantly increased in the M100 group compared with the ELV group (*P* < 0.05) (Figure 2(a)).

### 3.4. Changes in Spinogenesis in the Arc

**Figure 5:**
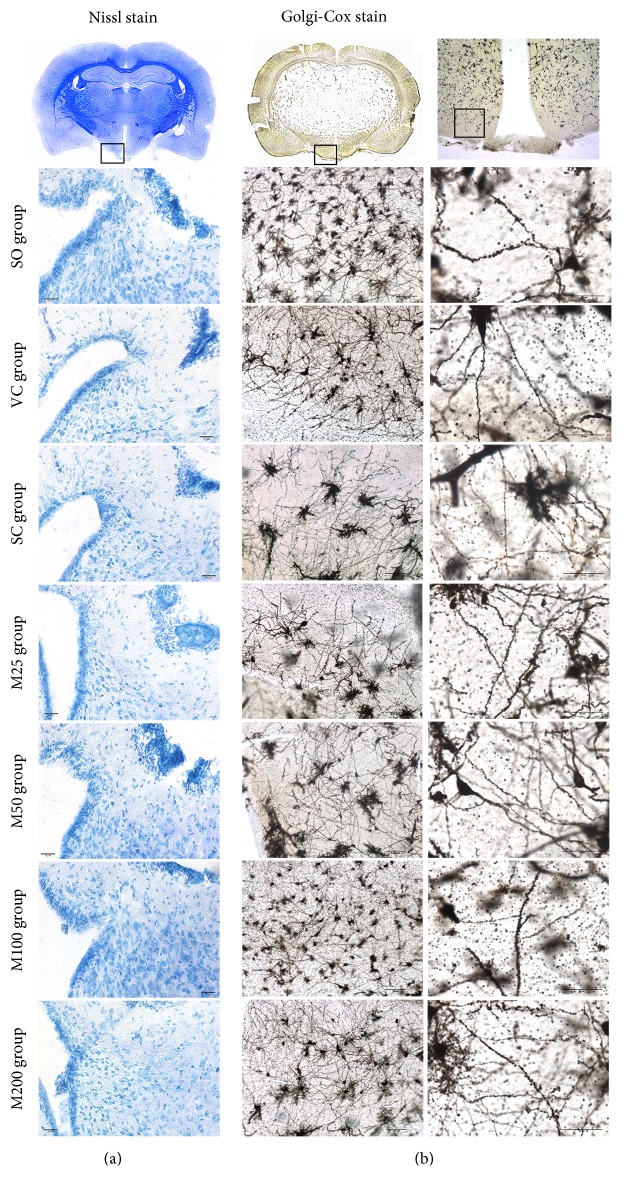
Nissl staining (a) and Golgi-Cox staining (b) of the neurons in the Arc of the experimental groups.

Microscopic analysis of Nissl-stained, 30 *μ*m brain tissue sections revealed that significant cell loss did not occur in the Arc of the hypothalamus after ELV establishment or MOP administration (Figure 5(a)).

Golgi-Cox staining (Figure 5(b), Table 3) indicated that ELV establishment significantly decreased the spine density and the percentage of thin spines in the Arc of the corresponding group compared with the Arc of the SO group (*P* < 0.05). As shown in Table 3, the spine density of the M25, M50, and M100 groups was significantly increased compared with that of the ELV group (*P* < 0.05). Moreover, the percentage of thin spines and the functional spine densities in the M25 and M50 groups (*P* < 0.05), as well as the functional spine density in the M100 group, were increased compared with the corresponding parameters in the ELV group. However, the percentage of thin spines was not changed in the M100 group compared with the ELV group. No changes in either spine density or the percentage of functional spines were observed in the M200 group, whose percentage of thin spines was lower than that of the other experimental groups (*P* < 0.05).

Synaptophysin expression was significantly decreased in the ELV and SC groups compared with the SO group (*P* < 0.05). However, synaptophysin expression was significantly increased in both the M50 group and M100 groups compared with the ELV group (*P* < 0.05).

## 4. Discussion

VCs are considered a major threat to male reproductive health; thus, studies aiming to identify treatments that can be administered either during the early phase of VC development or as prophylactic agents designed to prevent VCs in high-risk individuals are urgently needed.

As rat spermatogenesis is similar to human spermatogenesis [39], we established a classic ELV rat model to mimic the nutcracker phenomenon that affects humans. We intragastrically administered four different doses of MOPs immediately after the animal model was established to demonstrate the protective effects of MOPs on testes during the early stages of VC development.

Treatment with MOPs at doses ranging from 25 mg kg^−1^ to 200 mg kg^−1^ body weight was effective in increasing sperm quality and improving testis morphology to varying extents, indicating that MOPs may be effective in inhibiting reproductive damage during VC progression. Interestingly, serum GnRH secretion was not inhibited by the abovementioned increases in gonadotropin levels in the groups treated with 25 mg kg^−1^–100 mg kg^−1^ MOPs, suggesting that MOPs may stimulate GnRH secretion in the hypothalamus. However, the low serum GnRH levels noted in the M200 group may be attributable to the participation of sex hormones in a negative-feedback loop involving the hypothalamus.

The Kiss1/GPR54 pathway, a crucial excitatory regulator of GnRH neurons, has been recognized as a gatekeeper of reproduction. Kisspeptins bind to and activate GPR54, induce potent and durable depolarizing responses in GnRH neurons, and stimulate GnRH secretion via PLC-Ca^2+^ pathway activation. In rodents, Kiss1 neurons prominently reside in the Arc and preoptic area [40, 41]. Estrogen and T can downregulate Kiss1 expression in the Arc, leading to decreases in GnRH secretion. This is the mechanism through which sex hormones regulate GnRH production via negative-feedback loops [42]. The ME, the major region containing GnRH neuronal terminals, is located in the coronal portion of the Arc, which was selected as the plane of observation in this study. We performed micropunctures to collect tissue samples from the ME and other sections of the Arc to avoid biasing our results.

### 4.1. MOPs Promote GnRH Secretion by Upregulating the Kiss1/GPR54 Pathway

GnRH is released from nerve terminals in the ME into portal capillary vessels, and a population of GnRH neurons are scattered throughout the Arc [43]. We noted GnRH immunoreactivity in the ME and lateral region of the Arc and evaluated GnRH secretion in experimental animals by detecting GnRH levels in the Arc and ME.

Low doses (25 mg kg^−1^) of MOPs were effective in increasing hypothalamic GnRH levels but were not effective in protecting the seminiferous epithelium, as demonstrated by HE staining of the testes. Medium doses (50 mg kg^−1^) and medium-high doses (100 mg kg^−1^) of MOPs were effective in stimulating hypothalamic GnRH secretion, promoting spermatogenesis, and preserving germ cell function. These results indicate that MOPs may protect against VC-induced reproductive damage by preventing germ cell loss and stimulating GnRH secretion and that they may exert their effects on the hypothalamus before they exerts their effects on the testes. Additionally, GnRH synthesis in the hypothalamus was not inhibited by high serum GnRH levels and sex steroids in the M50 and M100 groups. These results may indicate that MOPs stimulate GnRH synthesis in the hypothalamus and thus induce subsequent increases in gonadotropin and T levels.

Administration of 200 mg kg^−1^ MOPs induced the production of extremely high levels of sex steroid hormones, which caused decreases in hypothalamic GnRH secretion and had detrimental effects on spermioteleosis, leading to sperm abnormalities. Thus, 50 mg kg^−1^–100 mg kg^−1^ MOPs may be the most effective and appropriate doses for attenuating the damage to the hypothalamus and testes associated with VC progression.

The Kiss1/GPR54 pathway is an essential, excitatory upstream regulator of GnRH neurons but is not essential for maintaining basal levels of circulating gonadotropins [42]. Kiss1 and GPR54 expression levels were downregulated after VC, possibly as a result of decreases in serum gonadotropin and T levels. Administering MOPs at doses between 50 mg kg^−1^ and 100 mg kg^−1^ effectively increased the expression of Kiss1 and GPR54 in the Arc, as well as the phosphorylation of Erk1/2 and p38, which participate in the Kiss1-GPR54 signaling cascade [44]. Thus, Kiss1/GPR54 pathway upregulation may be one of the mechanisms by which MOPs stimulate GnRH secretion. Additionally, our observation that MOPs upregulated the transcription factors that stimulate GnRH transcription may indicate that MOPs have direct stimulatory effects on GnRH synthesis, a phenomenon that needs to be assessed in future studies.

Furthermore, a subset of Kiss1 neurons in the Arc are responsible for the negative-feedback loop-mediated effects of sex steroid hormones. The decreases in Kiss1 expression in the Arc and the subsequent decreases in GnRH secretion in the M200 group may be attributable to the effects of high levels of T, which has been reported to downregulate Kiss1 mRNA expression in the Arc [45, 46]. Moreover, the high serum gonadotropin and T levels noted after the administration of 200 mg kg^−1^ MOPs may indicate that MOPs have stimulatory effects on either pituitary or Leydig cell secretory activity, a hypothesis that must be assessed in future studies.

### 4.2. MOPs Promote Spinogenesis and Spine Maturation in the ARC

The Golgi staining results indicated that MOP treatment at doses ranging from 25 mg kg^−1^ to 100 mg kg^−1^ significantly induced the formation of new spines in the Arc of ELV rats. MOPs (at a dose of 25 mg kg^−1^) initially increased the percentage of filopodia-shaped spines. Then, the percentage of mushroom-shaped spines increased with increasing doses (50 mg kg^−1^ and 100 mg kg^−1^) of MOPs. The shift in morphology suggests that MOPs promote spinogenesis and cause immature filopodial spines to mature into mushroom-shaped spines to form dendrites that process incoming synaptic signals.

Testosterone-induced spinogenesis has been documented in the hippocampus, and increases in spine density have been shown to be essential for synaptic plasticity and hippocampus-dependent working memory [47, 48]. We did not observe any correlations between serum T levels and filopodial spine percentages, perhaps because of the significant increases in the numbers of mushroom-shaped spines in the M50 and M100 groups. However, we noted a positive correlation between MOP doses and mushroom-shaped spine counts in the MOP-treated groups (at the doses ranging from 25 mg kg^−1^ to 100 mg kg^−1^). These results may indicate that MOPs stimulate spine maturation in the Arc of ELV rats. In addition, we found that treatment with MOPs at a dose of 200 mg kg^−1^ induced decreases in the numbers of filopodial spines and increases in the numbers of mushroom-shaped spines, which may indicate that high doses of MOPs have some negative effects on spinogenesis.

Among the MOP-treated groups, the M50 group had the highest percentage of filopodial spines, and the M100 group had largest number of mushroom-shaped spines. These results indicate that treatment with MOPs at doses of 50 mg kg^−1^ and 100 mg kg^−1^ promotes spinogenesis and spine maturation, respectively, in the Arc of ELV rats.

### 4.3. The Possible Mechanisms by Which MOPs Induce Sustained GnRH Secretion

The above changes in spine morphology may induce corresponding changes in synaptic plasticity, which is crucial for neuronal function. In this study, treatment with MOPs at doses ranging from 50 mg kg^−1^ to 100 mg kg^−1^ significantly upregulated synaptophysin expression, indicating that MOPs effectively enhanced neuronal synaptic plasticity in the Arc.

Kiss1 neurons residing in the Arc are responsible for receiving sex steroid hormone signals and triggering a negative-feedback loop by modulating Kiss1 secretion. The increases in the numbers of spines, the increases in synaptophysin expression, and the upregulations of Kiss1 and GnRH expression in rats treated with MOPs at doses ranging from 50 mg kg^−1^ to 100 mg kg^−1^ may be indicators of neuronal functional repair and improvements in the rats. Kiss1 neurons are the main neurons that reside in the Arc of rats; however, a small population of GnRH neurons is also located in the Arc. Thus, the stimulatory effects of MOPs on GnRH secretion may be closely related to the protective effects of Kiss1 and GnRH neurons on spine function.

Our observation that treatment with MOPs at doses ranging from 25 mg kg^−1^ to 100 mg kg^−1^ increased hypothalamic and serum GnRH levels, as well as sex steroid hormone levels, suggests that the negative-feedback loop-mediated effects of sex steroids on the hypothalamus may be partially inhibited by the above treatments. The gradual increases in Kiss1 expression noted in the M25, M50, and M100 groups indicated that sex steroid hormones failed to inhibit Kiss1 synthesis in these groups. These results, as well as the results of the testicular morphology and sperm analyses, suggest that the proper oral dose of MOPs for ELV rats is 50 mg kg^−1^ d^−1^–100 mg kg^−1^ d^−1^. The human-equivalent dose (HED), which was determined by a calculation based on body surface area, is 8 mg kg^−1^ d^−1^–16 mg kg^−1^ d^−1^. Thus, the equivalent daily dose of MOPs for a human weighing 70 kg is 0.6 g–1.1 g. As polysaccharides account for approximately 9% of the total weight of dried* M. Officinalis*, the corresponding treatment dose of* M. Officinalis* is 6.7 g–12.2 g, which is safe for humans, according to the results of studies regarding the use of* M. officinalis* in Chinese clinical practice.

In conclusion, MOPs were effective in protecting reproductive function in VC progression, as they promoted spermatogenesis and stimulated GnRH secretion. The stimulatory effects of MOPs on GnRH secretion, which is crucial for gonadotropin secretion and gonadal function, may be closely related to Kiss1/GPR54 pathway upregulation.

## Figures and Tables

**Table 1:** Quantitative real-time PCR primer sequences.

Gene	Primer sequence
GnRH	F: AACAATGCGTCTCTTGAGCA
	R: TACCCATATATAAGTGGGTCG
Oct-1	F: AACATTAAGTGAAGGCGCCGA
	R: AAATAATGGCCTCGATTAAGC
SCIP	F: ATGGCCACCACCGCGCAGTACCTGCCGC
	R: TCACTGCACTGAGCCGGGC
GPR54	F: GTTCCGCAGCTGTTTCCG
	R: GCATAGCTTCGAGGGTGCCA

**Table 2:** Sperm parameters and JSs of the experimental groups.

	Sperm count (×10^6^)	Abnormality (%)	Johnsen score
SO group	196.38 ± 13.64	9.83 ± 0.67	9.76 ± 0.13
ELV group	96.21 ± 9.11^a^	53.37 ± 7.89^a^	2.12 ± 0.17^a^
SC group	98.73 ± 8.99^a^	51.21 ± 6.36^a^	2.23 ± 0.14^a^
M25 group	121.37 ± 14.41^a^	42.24 ± 3.83^a^	6.87 ± 0.41^ab^
M50 group	168.71 ± 11.24^b^	21.23 ± 2.47^ab^	8.21 ± 0.32^ab^
M100 group	183.43 ± 18.21^b^	10.29 ± 0.87^b^	9.53 ± 0.31^b^
M200 group	162.38 ± 16.88^ab^	48.61 ± 4.29^a^	9.77 ± 0.18^ab^

^a^ *P* < 0.05: compared with SO group; ^b^*P* < 0.05: compared with ELV group.

**Table 3:** Spine density and maturity in the experimental groups.

	Number of neurons (/25 mm^2^)	Spine density (/50 *μ*m)	Thin spines (%)	Functional spines (/50 *μ*m)
SO group	8.13 ± 2.12	46.34 ± 4.71	47.33 ± 5.31	28.66 ± 4.94
ELV group	5.34 ± 1.71	22.32 ± 5.12^a^	23.37 ± 4.11^a^	13.31 ± 4.84^a^
SC group	5.17 ± 2.32	23.43 ± 4.37^a^	20.63 ± 3.33^a^	14.17 ± 3.33^a^
M25 group	5.44 ± 1.82	34.63 ± 5.17^ab^	33.33 ± 2.18^ab^	19.23 ± 3.18^ab^
M50 group	6.13 ± 1.74	37.22 ± 4.36^b^	42.12 ± 5.67^b^	25.88 ± 4.67^b^
M100 group	8.38 ± 2.11	45.58 ± 3.24^b^	23.13 ± 4.58^a^	34.87 ± 4.58^ab^
M200 group	7.26 ± 1.81	19.89 ± 4.11^a^	11.62 ± 3.11^a^	16.38 ± 3.11^a^

^a^ *P* < 0.05: compared with SO group; ^b^*P* < 0.05: compared with ELV group.
